# Workplace learning through peer groups in medical school clerkships

**DOI:** 10.3402/meo.v19.25809

**Published:** 2014-11-25

**Authors:** Calvin L. Chou, Arianne Teherani, Dylan E. Masters, Margo Vener, Maria Wamsley, Ann Poncelet

**Affiliations:** 1 Department of Medicine, University of California–San Francisco, San Francisco, CA, USA; 2 Department of Medicine, Veterans Affairs Medical Center, San Francisco, CA, USA; 3 Department of Medicine and Office of Medical Education, University of California–San Francisco, San Francisco, CA, USA; 4 School of Medicine, University of California–San Francisco, San Francisco, CA, USA; 5 Department of Family and Community Medicine, University of California–San Francisco, San Francisco, CA, USA; 6 Department of Neurology, University of California–San Francisco, San Francisco, CA, USA

**Keywords:** clinical education, workplace learning, peer continuity

## Abstract

**Purpose:**

When medical students move from the classroom into clinical practice environments, their roles and learning challenges shift dramatically from a formal curricular approach to a workplace learning model. Continuity among peers during clinical clerkships may play an important role in this different mode of learning. We explored students’ perceptions about how they achieved workplace learning in the context of intentionally formed or *ad hoc* peer groups.

**Method:**

We invited students in clerkship program models with continuity (CMCs) and in traditional block clerkships (BCs) to complete a survey about peer relationships with open-ended questions based on a workplace learning framework, including themes of workplace-based relationships, the nature of work practices, and selection of tasks and activities. We conducted qualitative content analysis to characterize students’ experiences.

**Results:**

In both BCs and CMCs, peer groups provided rich resources, including anticipatory guidance about clinical expectations of students, best practices in interacting with patients and supervisors, helpful advice in transitioning between rotations, and information about implicit rules of clerkships. Students also used each other as benchmarks for gauging strengths and deficits in their own knowledge and skills.

**Conclusions:**

Students achieve many aspects of workplace learning in clerkships through formal or informal workplace-based peer groups. In these groups, peers provide accessible, real-time, and relevant resources to help each other navigate transitions, clarify roles and tasks, manage interpersonal challenges, and decrease isolation. Medical schools can support effective workplace learning for medical students by incorporating continuity with peers in the main clinical clerkship year.

Medical students often struggle in their major clerkship year (1–4), in part because of the striking shift in expectations for how students learn between the pre-clerkship and clerkship years. Effective learning skills that a student has practiced in college and the pre-clerkship years, mainly emphasizing individual achievement in the classroom, do not translate into the clinical environment, where competing directives (e.g., patient care, resident learning, meeting regulatory standards, medical center and financial pressures, and research interests) do not directly focus on students’ learning (5). In addition, students’ roles and responsibilities are often unclear, expectations are not explicitly communicated, and clinical pressures on teams dictate that necessity and expediency determine selection of tasks and activities for students. Faculty and residents typically focus on teaching fundamental clinical knowledge and skills, not remembering to explicate the ‘hidden knowledge’ that students need to become effective team members because it is so routine and implicit in their practice (6).

Therefore, successful clerkship-based learning requires more than mastery of traditional clinical knowledge and skills. Students undergo a dramatic shift in role from formal curricular learning to learning in the workplace. ‘Workplace learning’ is a concept increasingly recognized for its fundamental importance in students’ professional development and involves three domains: ‘relationships’ within the practice community, the nature of work ‘practices’, and selection of ‘tasks’ and activities (7–10). Relationship factors include managing personalities, navigating the workplace with or without guidance and support, effective communication, and understanding roles. Work practice factors include workload and pace, mix of routine and nonroutine activities, resource constraints, and scheduling. Tasks include required knowledge and technical skill, complexity, speed, implicit knowledge, sequencing, and acuity. However, many of these expectations in clinical settings remain implicit, and third-year medical students (MS3s) on clerkships rarely receive formal orientation to methods of workplace learning, resorting to informal means of passing on this important information to each other (1, 9).

Continuity has gained increasing recognition as an important element in the core clinical training of medical students (11, 12) and can take several forms, including continuity with patients, teachers, and medical systems. At our institution, we constructed several structured ‘clerkship models with continuity’ (CMCs) with curricula focused explicitly on professional development and student-focused learning (13–15). We previously showed that in the context of these models, another element of continuity, continuity with peers, can help provide academic and social support for medical students in the core clerkship year (13). In this study, we sought to explore whether peer continuity, in the form of peer groups in traditional block clerkships (BCs) or in intentionally formed CMCs, also provides support for effective workplace learning.

## Methods

We received institutional review board approval for this qualitative study of peer relationships and their effects on workplace learning for MS3s in different clerkship models at our institution.

Participants were MS3s approximately halfway through their core clerkship year at a public medical school in academic year 2011–2012. Students elected to participate in BCs or in one of three different CMCs, each based at a different medical center. BCs took place at numerous sites including the three major medical centers where CMCs occurred, ranging in length from 4 to 8 weeks, provided clinical experiences in predominantly inpatient settings, and included a longitudinal ambulatory preceptorship intercalated throughout the year. Each year, 60% of students enroll in BCs. CMCs share similarities with each other including stable peer cohorts throughout their programs and regularly scheduled (typically weekly) continuity meetings of peer cohorts. One CMC was a yearlong longitudinal integrated clerkship based at the university medical center and integrated all core clerkships by assigning students to preceptors in each discipline (15). The other two CMCs were 6 months in length and occurred at other local major urban teaching hospitals (13, 14).

**Fig. 1:**
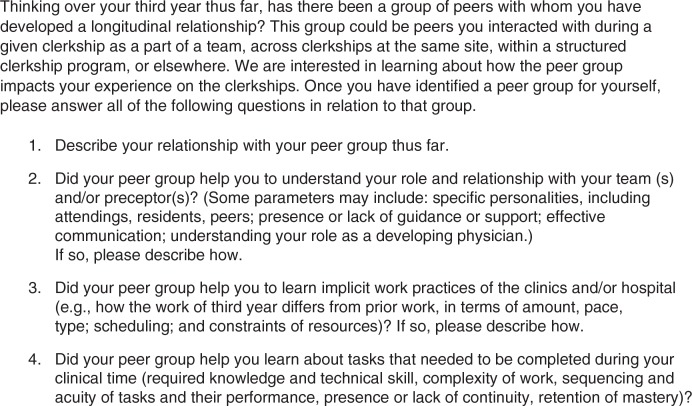
Workplace learning survey distributed to participants.

As part of a required reflection activity, we invited a random sample of students in the CMCs (*N*=29) and a convenience sample of students in BCs (*N*=44) to complete an anonymous survey of open-ended questions (Fig. 1) based on the workplace learning framework (7). We conducted content analysis using open and axial coding (16) to examine survey data. Using the constant comparative method (17), all investigators read eight surveys, two per program, to develop initial descriptive codes. We checked and refined these codes analyzing an additional two surveys per program. Upon reaching agreement, three investigators reviewed each remaining survey. Discussion among investigators reconciled analytic discrepancies.

## Results

In total, 54 (40% of the entire class, 74% of invited group) students completed the surveys. Of the 54 participants, 28 (97% of the CMC invited group) were CMC and 26 (59% of the BC invited group) were BC students.

### Peer learning groups in the clinical setting

CMC students almost always identified their CMC peer cohort as their core peer group; occasionally, they identified an additional peer group from earlier in medical school. CMC students perceived that ‘regular’ facilitated interaction structured throughout the CMC represented a key feature of their peer group. CMCs provided students an opportunity to form relationships with fellow CMC students, who became essential sources of social and academic support. BC students met occasionally with their peer group and voiced their wish to see their peers more often during the clerkship year. BC students identified their peer groups as students from the first 2 years (e.g., pre-existing educational track, basic science small groups), or students who fortuitously shared one or more clerkships. However, some BC students had difficulty finding peer groups.

Almost all students described their identified peer groups as sources of support, both socially and academically. Social support included ongoing friendships and forums for sharing anxieties about the clerkship year. Students in CMCs valued regular meetings where they worked closely together. They described developing greater cohesion as a team due to increased and more consistent contact. In addition, they noted the value of ‘frequently checking in with each other outside [weekly] meetings, to help each other navigate through various issues that come up during our rotations’. One student wrote of ‘occasional annoyances and conflicts, but overall it has been a very positive experience’. In contrast, students in BCs tended to meet on an *ad hoc* basis, ‘often due to busy schedules that do not usually coincide’. One BC student wrote of a lack of peer group relationships and ‘felt almost like a nomad … constantly trying to catch up’. BC students who occasionally fell together serendipitously in the same clerkship schedule did become closer and frequently checked in with each other.

Academic support themes included assistance with study skills, sharing learning materials, and transitions to new clinical experiences. Both CMC and BC students mentioned how peers reassured them as they struggled to understand exactly what to study by filling knowledge gaps, accessing and sharing learning materials, and providing an informal standard against which they could determine the appropriate balance between studying and clinical work.

### Workplace learning

#### Relationships

Peers in both BCs and CMCs provided anticipatory guidance that helped each other enter new clinical situations with a better understanding of their role. Peer groups oriented students to the culture of clinical settings and teams, students’ responsibilities on the team, and methods for efficient integration into a team. Students greatly valued learning from peers about what upcoming teams expected of them, including how to function effectively and how to interact with residents and faculty. One student stated,

Talking with peers helps me realize what kind of work we should be doing as MS3's and what is a reasonable patient load.

Some students felt that peer groups were particularly helpful for sharing information about roles and responsibilities across and within blocks and when working with multiple teams.

Peer groups also guided students on how best to communicate and maximize their learning time with supervising faculty and residents. Peers gave advice on individual supervisors’ expectations of students and how to handle those expectations. For example, peers gave advice on which supervisors were approachable, how to respond to feedback, and how to navigate difficult supervisor personalities while ‘normalizing’ stressful situations.

Peers also helped guide students about how to care for and communicate with a range of patients:

If I share a story about a patient I am struggling to communicate with, they would say, ‘you could phrase it this way’, or ‘this is what has worked for me’.

A minority of students from both BCs and CMCs felt their peer group was not important to understanding their roles and responsibilities. These students felt that their peers from the first 2 years of medical school and longitudinal preceptors were more helpful in this regard.

#### Practices

For both BC and CMC students, implicit practices most prominently consisted of helping with transitions between rotations. Areas that students discussed with each other encompassed expectations about workload and hours, suggestions about managing daily schedules and developing timelines for studying for end-of-clerkship exams, and methods of accomplishing clerkship requirements. Some mentioned that peers could help direct attention to talks or extra learning sessions about which individual students may not have been formally notified.

CMC students emphasized the importance of comparing themselves with their peers in the workplace. These comparisons had both positive and negative dimensions. Some reported that comparing their achievements and skills with peers, both favorably and unfavorably, provided a ‘yardstick’ with which to measure their own progress. Sometimes this motivated students, inspiring them to work harder. However, it also caused some feelings of insecurity: students felt that they did not know as much as their peers, heightening awareness that clerkship grades typically incorporate comparisons with peers.

It can definitely serve as motivation … but it also leads to insecurity and always feeling acutely aware of how little you know compared to the third year standing next to you.

There were practices that students felt that they learned better on their own or from supervising residents or faculty, rather than from peers.

Everyone does not come in with the same level of skills, so it really becomes a personal goal to figure out what skills need to be developed.

Students also commented how independent learning grew from the fact that different preceptors or medical teams held different expectations for each student.

#### Tasks

Both groups of students appreciated peer support in understanding specific skills and tasks, such as navigating the electronic medical record, performing discipline-specific physical exam skills and procedures, or accomplishing work related to patient care.

I shared with my classmates what resources were available for that rotation and how to work the system at the hospital. Many times, some of the small things like finding patient charts or finding other pieces of information are difficult but my classmates made sure to help me with this.

Another student stated:

It was always handy to have people to ask about how do I find respiratory therapy or who do I call to place certain orders.

Similar to the theme of comparing oneself with one's peers in the ‘practices’ section above, some students stated that working with peers helped gauge their own level of knowledge and skills, expanding their own responsibilities as a medical student to improve learning. Both groups of students found peer support to provide an external check on knowledge base and completion of required tasks for the clerkship.

In our discussions, I often found gaps in my knowledge that I didn't realize I had.

Unique to CMC students was support in building patient panels and enabling patient continuity as well as obtaining important clinical experiences and improving workflow and efficiency.

I think we all helped each other figure out how best to learn in our various settings, keep continuity with patients, and improve our workflow.

## Discussion

We show that MS3s seek out their own workplace-based peer groups (WPGs) during clerkships, and that these peer groups substantially help with effective workplace learning. We coin the term ‘workplace-based peer groups’ for these groups that emerge from both informal structures (site continuity for 6–12 months in CMCs, or fortuitously for BC students) and formal meetings (weekly facilitated groups in CMCs). We also distinguish these WPGs from communities of clinical practice, the clinical teams into which students integrate throughout the year. Whereas communities of clinical practice also encompass faculty and resident supervisors, allied health professionals, and patients (5), these parallel and separate WPGs revolve around student learners as they discover together how to reflect about and to legitimize their experiences in their coexisting communities of practice. Overall, the CMCs support the formation of effective WPGs, which in turn augment the workplace learning that takes place as students transition from one clinical arena to another. Students in traditional BCs, on the other hand, are left to develop *ad hoc* groups on their own. At least one student reported not finding a WPG, a potentially significant disadvantage given that WPG students described enriching resources that supported them throughout clerkships.

Our study deepens prior work examining challenges that students experience when moving from pre-clerkship activities to clerkships (6, 8, 9, 18–22). We found three ways in which WPGs provided support through those challenges. First, WPGs avail third-year students to real-time, accessible, and relevant resources to facilitate their transformation into seasoned and effective workplace learners. WPGs therefore confer an advantage over an alternative prevalent in many schools, the intercalation of periodic intersessions throughout the clerkship year where students can reflect on challenges and experiences in professional development (11, 22). Second, a common struggle for students entering immersive clinical practice is not merely adapting to the pace and tasks of a vastly different workplace, but to achieve a true sense of belonging and an ability to contribute (23, 24). Although students do not rely on WPGs to provide all workplace learning needs, WPGs confer the supported participation that enables students to share best practices with each other on how to integrate into their communities of clinical practice (9, 25). Finally, here and in prior studies, students appreciated the increased social support provided by their WPGs (14, 15), a key feature of medical student well-being. It remains to be seen whether students truly become less burnt-out and more resilient as a result of participating in WPGs (26, 27). Future work could also characterize the continuity of these groups beyond clerkship training as another measure of the value of social support.

WPGs have their challenges: students mentioned competition and personality differences as sources of difficulty. In any social learning environment with an evaluative component, it is natural that competition should arise. Interestingly, students stated they sometimes learned more in a competitive environment, both in workplace-based practices and tasks. Though not much is known about the nature of competition, a recent study invoked social comparison theory to suggest that comparisons with other students influence a student's self-efficacy of clinical learning (28). Future studies should explore the influence of WPGs on positive and negative aspects of competition, as well as the deeper influences of social comparison on peer learning.

Limitations to this study include its single-institution and single-year design. We derived themes from a written survey, with no further probing or follow-up to comprehend deeper meanings. Students who choose CMCs might be students who thrive in social learning settings, although many BC students also spoke of the benefits of social learning. We emphasized the similarities between CMCs with site and peer continuity; however, there are clear differences between the programs, one of which is a longitudinal integrated clerkship (15), that merit further research.

We show that WPGs provide peer support for navigating transitions between clerkships, discovering central roles and tasks as they transform into workplace learners, and sharing advice on challenging situations. Though WPGs can allow expression of challenging dynamics such as competition, the advantages of providing supportive spaces for students to work together on their professional development appear to outweigh those risks. Regardless of format, workplace-based peer learning in groups will occur, with more well-connected students accessing more resources, potentially leading to disparities in learning. With minimal redesign effort (14, 29), medical schools can decrease these differences in clerkships by incorporating continuity with peers, whether through CMCs, extensions of previously established learning communities explicitly in workplace settings, or other novel structures.
